# Tissue Kallikrein Mediates Pro-Inflammatory Pathways and Activation of Protease-Activated Receptor-4 in Proximal Tubular Epithelial Cells

**DOI:** 10.1371/journal.pone.0088894

**Published:** 2014-02-21

**Authors:** Wai Han Yiu, Dickson W. L. Wong, Loretta Y. Y. Chan, Joseph C. K. Leung, Kwok Wah Chan, Hui Yao Lan, Kar Neng Lai, Sydney C. W. Tang

**Affiliations:** 1 Department of Medicine, Queen Mary Hospital, The University of Hong Kong, Hong Kong; 2 Department of Pathology, Queen Mary Hospital, The University of Hong Kong, Hong Kong; 3 Department of Medicine and Therapeutics, and Li Ka Shing Institute of Health Sciences, The Chinese University of Hong Kong, Hong Kong; University of British Columbia, Canada

## Abstract

Tissue kallikrein (KLK1) expression is up-regulated in human diabetic kidney tissue and induced by high glucose (HG) in human proximal tubular epithelial cells (PTEC). Since the kallikrein-kinin system (KKS) has been linked to cellular inflammatory process in many diseases, it is likely that KLK1 expression may mediate the inflammatory process during the development of diabetic nephropathy. In this study, we explored the role of KLK1 in tubular pro-inflammatory responses under the diabetic milieu. Recombinant KLK1 stimulated the production of inflammatory cytokines in PTEC via the activation of p42/44 and p38 MAPK signaling pathways. Molecular knockdown of endogenous KLK1 expression by siRNA transfection in PTEC attenuated advanced glycation end-products (AGE)-induced IL-8 and ICAM-1 productions *in vitro*. Interestingly, exposure of PTEC to KLK1 induced the expression of protease-activated receptors (PARs). There was a 2.9-fold increase in PAR-4, 1.4-fold increase in PAR-1 and 1.2-fold increase in PAR-2 mRNA levels. Activation of PAR-4 by a selective agonist was found to elicit the pro-inflammatory and pro-fibrotic phenotypes in PTEC while blockade of the receptor by specific antagonist attenuated high glucose-induced IL-6, CCL-2, CTGF and collagen IV expression. Calcium mobilization by the PAR-4 agonist in PTEC was desensitized by pretreatment with KLK1. Consistent with these *in vitro* findings, there was a markedly up-regulation of tubular PAR-4 expression in human diabetic renal cortical tissues. Together, these results suggest that up-regulation of KLK1 in tubular epithelial cells may mediate pro-inflammatory pathway and PAR activation during diabetic nephropathy and provide a new therapeutic target for further investigation.

## Introduction

Diabetic nephropathy (DN) is a chronic, progressive renal disease that develops in approximately one third of people with diabetes. It is also the leading cause of end-stage renal disease (ESRD) in developed countries. In addition to pathognomonic features of glomerular hypertrophy, basement membrane thickening and mesangial matrix expansion, increasing evidence from numerous experimental and clinical studies of DN has demonstrated the participation of different inflammatory molecules and pathways. These data indicate that tubulointerstitial inflammation plays a critical role in the progression of DN to ESRD [1]–[3]. Inflammatory cytokines, mainly interleukin 1(IL-1), interleukin 6 (IL-6), interleukin 8 (IL-8) and tumor necrosis factor α (TNFα) are involved in the development of and progression to interstitial fibrosis in both diabetic patients and experimental models [4], [5]. Up-regulation of intercellular adhesion molecule 1 (ICAM-1) and chemokine (C-C motif) ligand 2 (CCL-2) leads to inflammatory cell recruitment and activation in the diabetic kidney [6]. In addition to cytokine production, induction of growth factors, such as insulin-like growth factor 1 (IGF-1), hepatocyte growth factor (HGF), transforming growth factor β (TGFβ) and connective tissue growth factor (CTGF) in proximal tubules enhances the activation and proliferation of myofibroblasts and collagen deposition, eventually resulting in interstitial fibrosis [7], [8]. In our previous studies, we have shown that high glucose (HG) stimulates IL-6 and CCL-2 expression via PKC activation and ERK1/2 signaling in cultured proximal tubular epithelial cells (PTEC) [9]; and advanced glycated end-products (AGE) and their carbonyl intermediates induce tubular IL-8, ICAM-1 expression via NFκB, ERK1/2 and STAT-1 signal transduction [10], [11]. These data suggest that HG, AGE and their intermediates play a pivotal role in the pathogenesis of diabetic tubular inflammation.

In fact, renal dysfunction correlates better with tubular and interstitial lesions than with glomerular changes [12]. Proximal tubules have been identified to be responsible for the change in urinary albumin excretion, which is independent of glomerular permeability changes during the very early stage of DN [13]. Other studies have also demonstrated the correlation between the induction of inflammatory markers and the degree of albuminuria prior to the development of fibrosis. The current therapeutic approaches have not been successful in preventing the progression of DN to ESRD. Thus, understanding the mechanisms of the underlying inflammatory pathways in DN is an important prerequisite for the development of more effective therapeutic strategies for the prevention of this relentless attrition of renal function in DN.

The kallikrein-kinin system (KKS) has been associated with inflammation, coagulation, pain and vascular permeability through the generation of kinins. Tissue kallikrein (KLK1), one of the components of KKS, is a serine protease that cleaves low molecular weight kininogen into kinin, which exerts the biological functions through kinin receptor, B1R and B2R signaling [14], [15]. We have previously shown that KKS is involved in the pathogenesis of DN. High glucose induced KLK1 and B2R expression in cultured PTEC and in human proximal tubules of the diabetic kidney [9]. Further *in vivo* data showed that treatment of the diabetic *db/db* mice with icatibant, a B2R antagonist, partially attenuated proteinuria and histological lesions in renal tissues [16]. In addition, the deletion of B2R protected against the development of streptozotocin (STZ)-induced DN [17]. These results suggested that tubular KLK1 expression may play a deleterious role during DN.

Although most of the biological functions of KLK1 are mediated by kinin receptor signaling, recent studies suggest that KLK1 may also activate protease activated receptors (PARs) in inflammatory and cardiovascular diseases [18], [19]. PARs are a subfamily of G protein-coupled receptors that are activated or inhibited by serine protease to expose a tethered ligand that binds to the receptor for signal transduction. There are four known members in the family, in which PAR-1 and PAR-3 are activated mainly by thrombin, PAR-2 is activated by trypsin and PAR-4 is activated by both enzymes [19], [20]. Other enzymes of the coagulation cascade such as tissue factors VIIa/Xa and activated protein C are also shown to be regulators of PARs [21]. Activation of the coagulation cascade occurs in the course of diabetic mellitus that affect fibrinolysis, platelet and endothelial functions. Fibrin deposition is commonly observed in tubulointerstitial damage of DN. PAR-2 expression was increased in proximal tubuli in IgAN nephropathy [22] and there was an up-regulation of PAR-1 expression in experimental diabetic glomerulosclerosis [23]. All these data indicated that the coagulation system may also play an important role in renal damage.

In this study, we investigated the role of KLK1 in tubular pro-inflammatory responses in cultured human PTEC and examined the role of PAR-4 activation in KLK1-mediated signaling in the development of DN.

## Materials and Methods

### Ethics Statement

The use of archival renal tissue for this study was approved by the Research Ethics Committee/Institutional Review Board of the University of Hong Kong/Hospital Authority Hong Kong West Cluster. The Institutional Review Board waived the need for consent for using these specimens.

### Reagents

Renal epithelial cell basal medium (REBM) and growth supplement were from Lonza Walkersville, MD. Recombinant human kallikrein (KLK1) was from ProSpec, Israel. PAR-4 agonist (AYPGKF-NH_2_) and PAR-4 antagonist (tcY-NH_2_) were from Tocris Bioscience, Ellisville, MO. AGE-BSA and D-glucose were from Sigma-Aldrich, St. Louis, MO. SYBR Green Master Mix was from Applied Biosystems, Carlsbad, CA. Lipofectamine™ 2000 and TRizol reagent were from Invitrogen, Carlsbad, CA.

### Renal Biopsies

Archival renal biopsies obtained from 5 patients with biopsy-proven DN (mean age of patients: 55; mean DM duration: 6.2 yrs; mean HbA1C: 7.2%; mean serum creatinine: 464 µmol/L; mean proteinuria: 5.80 g/24 h) were selected for this study. Normal portions of renal tissues removed from 5 archival nephrectomy specimens for the treatment of renal carcinoma (mean age of patients: 61; mean serum creatinine: 87.8 µmol/L) were used as control.

### Cell Culture

Human renal PTEC (Lonza) were cultured REBM with growth supplement at 37°C in 5% CO_2_ and 95% air. In all experiments, cells were used between passages 2–4 and were growth-arrested in serum free medium for 24 h before stimulation. Confluent, growth arrested PTEC were incubated in serum free medium containing 10–100 nM recombinant KLK1, 100–200 µM selective PAR-4 agonist (AYPGKF-NH_2_), 30 mM HG or 500 mg/ml AGE-BSA. To investigate whether HG/KLK1-induced cytokine expression was PAR-4 dependent, cells were pre-treated with 10 µM selective PAR-4 antagonist (tcY-NH_2_) for 1 h prior to HG/KLK1 stimulation.

### Gene Silencing by siRNA Transfection

PTEC were transfected with KLK1 or negative control of Silence®Select Pre-designed siRNA (Applied Biosystems) using Lipofectamine™ 2000 according to manufacturer’s instructions. The knockdown efficiency of KLK1 was determined by real-time PCR and Western Blot analysis. After 24 h transfection, cells were starved with serum free medium prior to AGE treatment.

### RNA Extraction and Real-time PCR Analysis

Total RNAs were isolated from PTEC using TRizol reagent. Two micrograms of total RNAs were reverse transcribed to cDNA and were amplified using SYBR Green Master Mix. The mRNA expression was analyzed by ABI 7500 Real-Time PCR System (Applied Biosystems). Primer sequences used were: IL-6, forward-ATG AAC TCC TTC TCC ACA AG and reverse-TGT CAA TTC GTT CTG AAG AG; CCL-2, forward-GAT CTC AGT GCA GAG GCT CG and reverse-TGC TTG TCC AGG TGG TCC AT; IL-8, forward-GTG CAG TTT TGC CAA GGA GT and reverse-TAA TTT CTG TGT TGG CGC AG; ICAM-1, forward-GGC CTC AGT CAG TGT GA and reverse-AAC CCC ATT CAG CGT CA; TGFβ forward-CAC GTG GAG CTG TAC CAG AA and reverse-GAA CCC GTT GAT GTC CAC TT; CTGF, forward-GGA AAA GAT TCC CAC CCA AT and reverse-TGC TCC TAA AGC CAC ACC TT; PAR-1, forward-GCC AGA ATC AAA AGC AAC AAA and reverse-TCA TTT TTC TCC TCA TCC TCC; PAR-2, forward-TGC TAG CAG CCT CTC TCT CC and reverse-CCA GTG AGG ACA GAT GCA GA; PAR-3, forward-GCT CAT CCT CTG CCT TCC and reverse-AAG TTA ATG GGC TTT CCT GC; PAR-4, forward-GGG TCC CTT CCC CCA CTT and reverse-GAC AGT TGT AAC AAC CCT ATT TCC AAA; KLK1, forward-GCC AAG CAG ACG AGG ACT AC and reverse-TTT GAG GTC CAC ACA CTG GA. Relative gene expression was obtained after normalization with β-actin, and followed by comparison to medium control using SDS software (Applied Biosystems).

### Calcium Mobilization

Intracellular calcium measurement was performed using Fluo-4 direct™ calcium assay kit (Invitrogen). Cells were grown at 2×10^4^ per well in 96-well cell culture plates for 24 h (Corning Costar, Corning, NY) and then incubated with equal volume of 2X Fluo-4 direct™ calcium reagent loading solution (Fluo-4 containing Hanks’ balanced salt solution, 20 mM HEPES, 5 mM probenecid, pH 7.3) for 1 h at 37°C. Fluo-4 fluorescence signal of labeled cells was measured using FLUOStar Omega plate reader (BMG labtech, Germany) for excitation at 485 nm and emission at 520 nm. Baseline signal was recorded for 10 sec, followed by the initiation of calcium mobilization with the addition of recombinant KLK1 (50–200 nM) and PAR-4 agonist AYPGKF-NH_2_ (100–300 µM) using the onboard reagent injector. For cross desensitization study, fluo-4-labeled cells were pretreated with KLK1 (100–200 nM) for 10 min prior to fluorescence measurement.

### Detection of Cytokines by ELISA

For cytokine expression analysis, culture medium was collected after 48 h treatment. IL-6, CCL-2, IL-8 and ICAM-1 protein levels were quantified using commercial kit (PeproTech, Rocky Hill, NJ) according to manufacturer’s instructions. The detection sensitivity range is 24–1500 pg/ml for IL-6, 16–1000 pg/ml for CCL-2, 8–1000 pg/ml for IL-8 and 23–3000 pg/ml for ICAM-1.

### Western Blot Analysis

Cells were lysed with lysis buffer containing protease inhibitor cocktails (Sigma-Aldrich) as described previously [9],[24]. Equal amount of proteins were resolved in 12% SDS-PAGE gel and transferred to PVDF membrane (Millipore, Bedford, MA). The membranes were incubated overnight with antibodies against PAR-1, PAR-2, PAR-3, PAR-4, CTGF (Santa Cruz Biotechnology, Santa Cruz, CA), Col IV, KLK1 (Abcam, Cambridge, UK), phosphor-p42/44 MAPK, p42/44 MAPK, phosphor-p38 MAPK and p38 MAPK (Cell Signaling Technology, Beverly, MA) in 5% non-fat milk, and subsequently incubated with peroxidase conjugated secondary antibodies (Dako, Carpinteria, CA). The immunocomplex was visualized with ECL prime chemiluminescence (GE Healthcare, Buckinghamshire, UK) using ChemiDoc XRS+ system (Bio-Rad, Hercules, CA). Quantification of protein bands was performed by the ImageJ program (NIH, Bethesda, MD) and was normalized to actin level.

### Immunohistochemistry

Expression of PARs was examined on paraffin-embedded human renal tissue section (4 µm) by immunohistochemical analysis. Briefly, deparaffinized and rehydrated sections were subjected to microwave-based antigen retrieval in 10 mM citrate buffer solution (pH 6) for 10 min, followed by quenching in 1% hydrogen peroxide solution for 10 min. The sections were then blocked with 2% BSA blocking buffer for 1 h and stained overnight with antibodies against PAR-1, PAR-2, PAR-3 and PAR-4 (Santa Cruz Biotechnology). The stained sections were further incubated with peroxidase conjugated secondary antibodies (Dako). The immunocomplex were visualized using DAB substrate from Envision Plus system (Dako). All sections were counterstained with hematoxylin before mounting. PARs staining were scored on 0–5 scoring system (0 = no cortical staining; 1 = <10% cortical staining; 2 = 10–20% cortical staining; 3 = 20–40% cortical staining; 4 = 40–60% cortical staining; 5 = >60% cortical staining) and were quantified by Image Pro Plus 6.0 software (Media Cybernetics, Silver Spring, MD). Data were expressed as integrated optical density (IOD).

### Statistical Analysis

All data were expressed as mean±SD from three independent experiments. Difference between multiple groups was evaluated by one-way ANOVA with Bonferroni’s comparison using GraphPad Prism, version 4 (GraphPad Software, San Diego, CA). Data were considered statistically significant at p<0.05.

## Results

### Tissue Kallikrein (KLK1) Induced Pro-inflammatory Cytokine Expression in PTEC via MAPK Signaling Pathway

**Figure 1:**
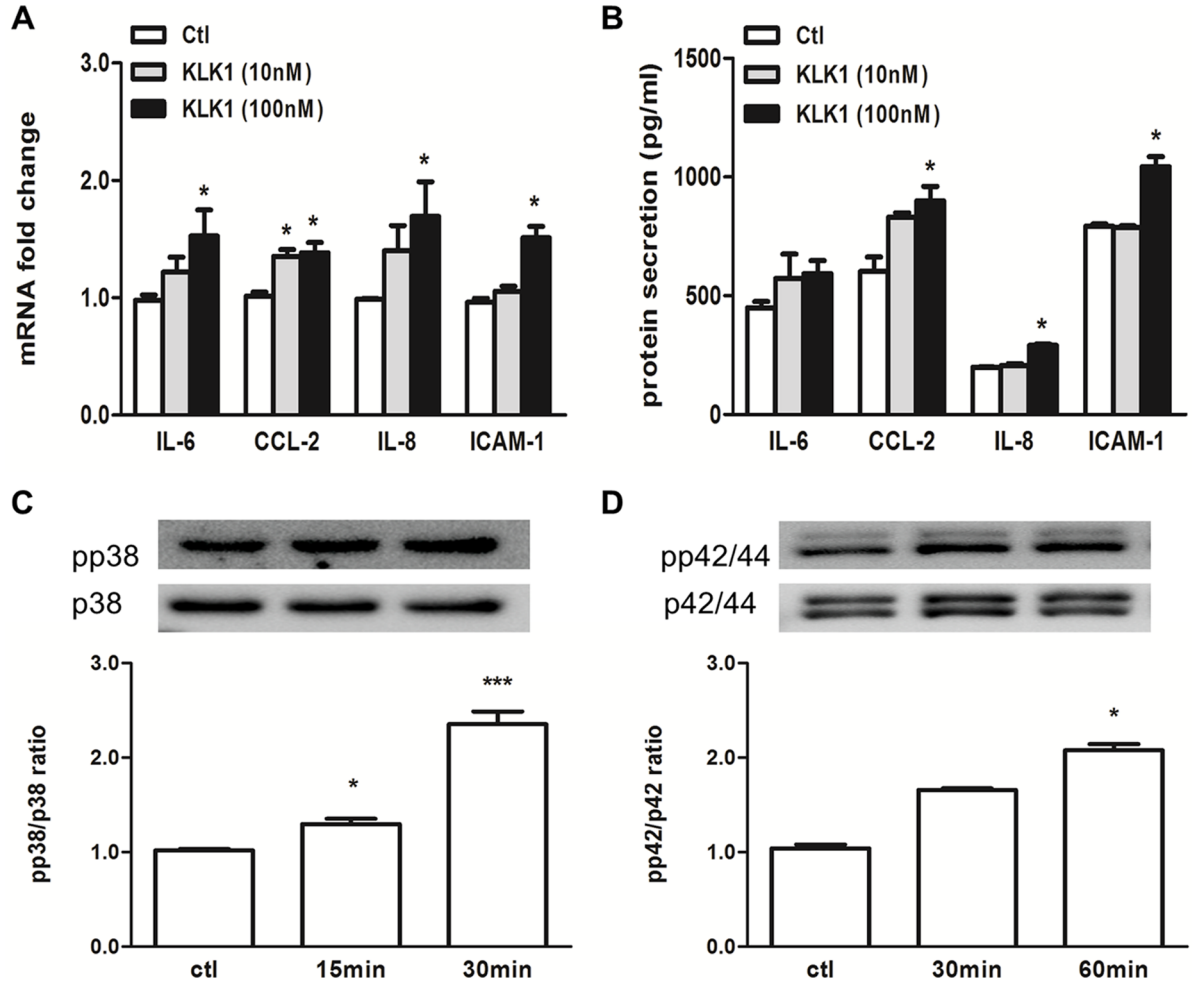
Recombinant KLK1 induced both cytokine expression and MAPK activation in tubular cells. PTEC was incubated with 10-time PCR and ELISA respectively. KLK1 increased IL-6, CCL-2, IL-8 and ICAM-1 mRNA (A) and protein (B) expression. PTEC was incubated with 100 nM KLK1 for the indicated duration, and expression of MAPK signaling molecules was detected by Western blot analysis. KLK1 increased phosphorylated p38 (C) and phosphorylated p42/44 (D) MAPK signaling pathways. *p<0.05; ***p<0.001 compared with control.

We have previously reported that KLK1 expression was up-regulated in the proximal tubules of human diabetic kidney and induced *in vitro* by HG in PTEC [9]. Here, we investigated the pro-inflammatory effect of KLK1 in cultured PTEC. Recombinant KLK1 at a dose of 100 nM significantly up-regulated the mRNA and protein expression of IL-6, CCL-2, IL-8 and ICAM-1 (Fig. 1A & 1B). KLK1 also activated phosphorylation of p38 and p42/44 MAPK signaling proteins (Fig. 1C & 1D).

### KLK1 Mediated AGE-induced Pro-inflammatory Responses in PTEC

**Figure 2:**
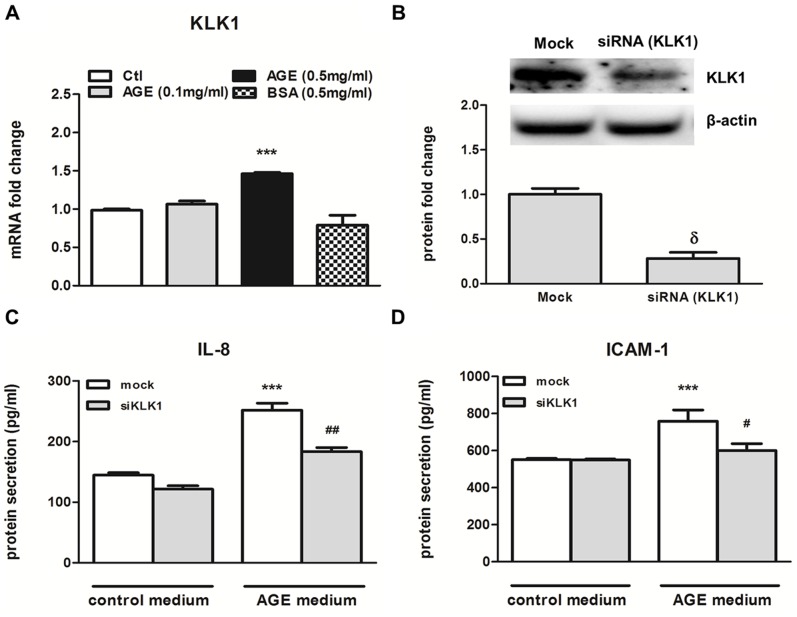
KLK1 mediated AGE-BSA induced IL-8 and ICAM-1 expression in PTEC. Cells were incubated with 0.1 or 0.5/ml AGE-BSA for 6 h, and gene expression was determined by real-time PCR analysis. AGE induced KLK1 mRNA expression in PTEC (A). Cells were transfected with KLK1-specific siRNA, and the endogenous protein level was determined by Western blot analysis (B). Transfected cells were incubated with 0.5 mg/ml AGE for 48 h, and protein expression of cytokine was detected in culture medium by ELISA. AGE -induced IL-8 (C) and ICAM-1 (D) protein expression was inhibited by KLK1 gene silencing. ***p<0.001 compared with control, ^δ^P<0.05 compared with mock transfection and ^#^p<0.05; ^##^p<0.01 compared with mock transfection incubated with AGE.

AGE stimulated expression of IL-8 and CCL-2 in renal tubular cells [10], these chemokines recruit leukocyte infiltration into the renal interstitial space, leading to the development of DN. Since KLK1 can stimulate the production of cytokines in PTEC, we next examined whether KLK1 participated in AGE-induced pro-inflammatory phenotype of PTEC. As shown in Fig. 2A, AGE (0.5 mg/ml), but not the equivalent dose of BSA, stimulated the transcript of KLK1 in PTEC. To study the role of KLK1 on AGE-induced cytokine production, cells were transfected with control or KLK1 siRNA, and then subjected to AGE stimulation. Western blot analysis showed that transfection with KLK1 siRNA significantly decreased endogenous KLK1 protein expression in PTEC (Fig. 2B) when compared with that of control siRNA. Knockdown of KLK1 attenuated AGE-induced IL-8 and ICAM-1 secretions in PTEC (Fig. 2C & 2D). These data suggested that endogenous KLK1 plays a role in mediating AGE-induced pro-inflammatory responses.

### Increased Expression of Protease-Activated Receptor 4 (PAR-4) by KLK1 and HG Stimulation

**Figure 3:**
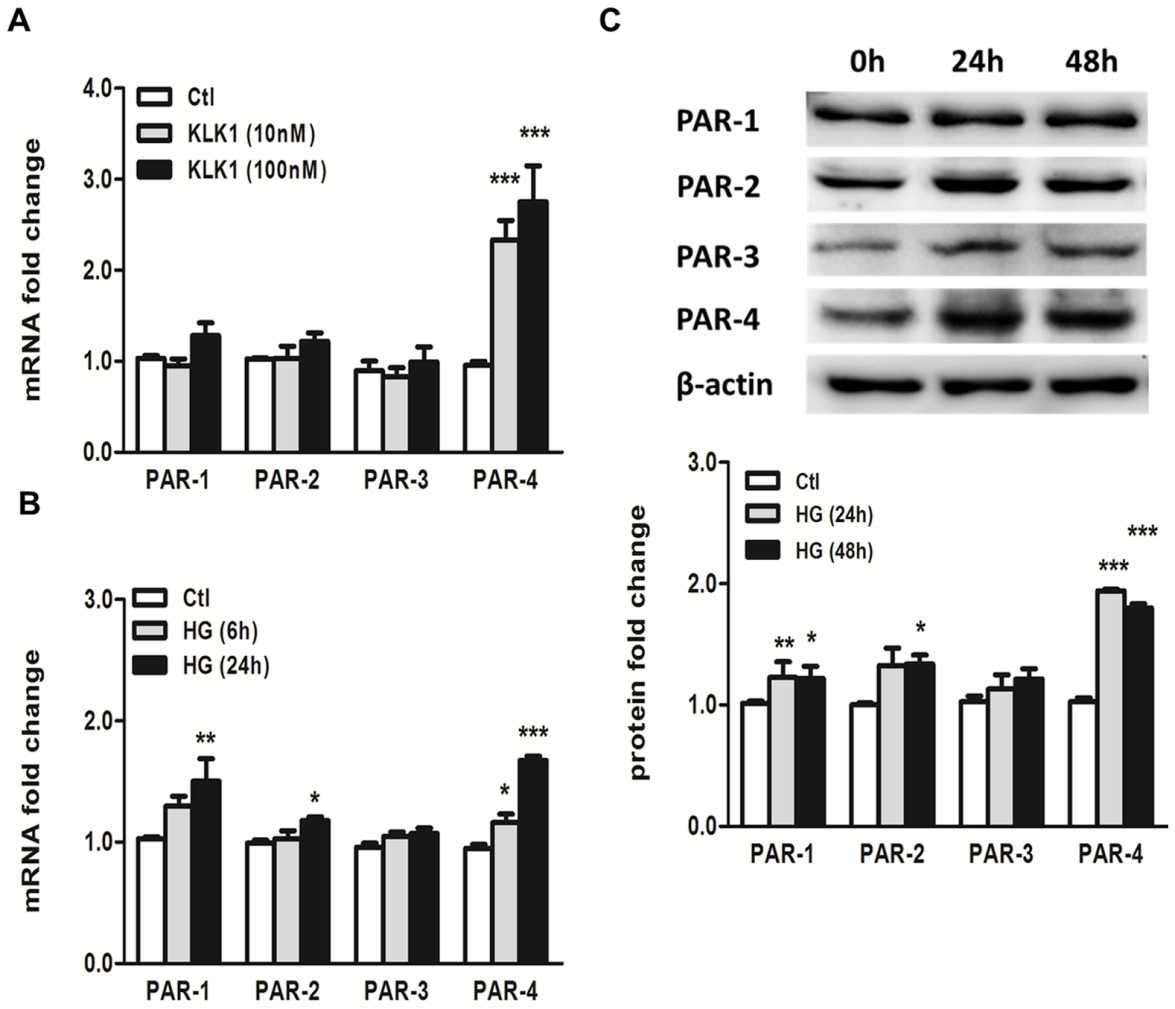
Increased expression of PAR-4 by KLK1 and HG in PTEC. Cells were incubated with 10-time PCR analysis. KLK1 induced PAR-4 mRNA expression in a dose-dependent manner (A). Cells were incubated with 30 mM HG for the indicated duration, and gene expression of PARs was determined by real-time PCR analysis. HG induced PAR-1, PAR-2 and PAR-4 mRNA expression (B) and protein expression (C) in a time-dependent manner. *p<0.05; **p<0.01; ***p<0.001 compared with control.

Recent studies showed that KLK1 can directly stimulate cell proliferation and migration through protease-activated receptors (PARs) [25], [26]. We next explored the participation of PARs in mediating the pro-inflammatory effect of KLK1 in PTEC. Real-time PCR analysis showed a marked induction of PAR-4 mRNA expression by 2.4 folds and 2.9 folds with 10 nM and 100 nM KLK1 stimulation, respectively (Fig. 3A). There was no significant change in PAR-1, PAR-2 and PAR-3 mRNA expression under the same condition. In addition to KLK1, incubation of PTEC with HG (30 mM) also increased PAR-1, PAR-2 and PAR-4 mRNA expression by 1.5 folds, 1.2 folds and 1.8 folds respectively, when compared to control (Fig. 3B). Similarly, the up-regulation of PAR-1, PAR-2 and PAR-4 proteins by HG was demonstrated by Western blot analysis (Fig. 3C) after 24-h and 48-h incubation.

### Increased Tubular PAR-2 and PAR-4 Expressions in Human Renal Biopsies from Patients with Diabetic Nephropathy (DN)

**Figure 4:**
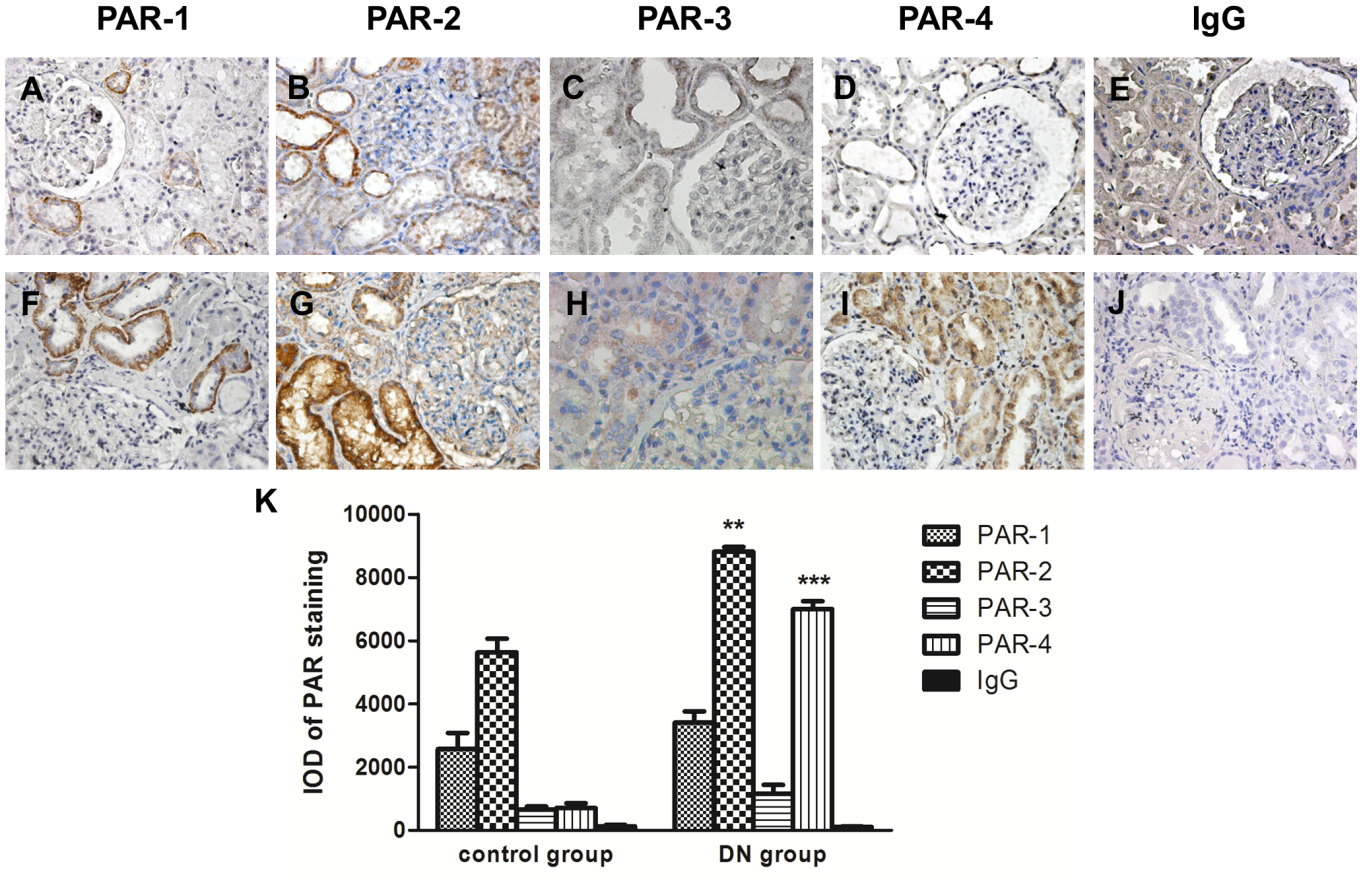
Representative immunohistochemical staining of PAR-1, PAR-2, PAR-3 and PAR-4 in human renal biopsies. Expression of PARs was demonstrated in renal tissue from patients with biopsy proven diabetic nephropathy (F–I) and non-diabetic control subjects (A–D). Isotype-matched negative control was shown in diabetic nephropathy section (J) and non-diabetic control (E). Magnification: 400X. Quantitative analysis of PAR staining by Image Pro Plus 6.0 software (K); IOD, Integrated Optical Density. **p<0.01; ***p<0.001 compared with control.

As PAR expression has been implicated in the pathogenesis of many inflammatory diseases [27]–[30] and some renal disorders [22], [23], we evaluated the expression of all PARs in renal biopsies from both DN patients and non-diabetic control subjects by immunohistochemical staining (IHC). Table 1 showed the clinical data of the study subjects and the intensity of PAR immunostaining in their kidney tissues. In normal subjects, mildly positive staining for PAR-2 was observed in the renal cortex, mostly in vascular and tubular cells (score 1–2), whereas only little staining was detected in the glomerulus (Fig. 4B). In DN biopsies, there was intense PAR-2 expression in the proximal tubules (score 2–4) and to a lesser extent in the glomeruli (Fig. 4G). In contrast, PAR-1, PAR-3 and PAR-4 (score 0–1) staining was barely detectable in normal renal cortex (Fig. 4A, 4C & 4D). Strong staining for PAR-4 (score 2–4) was detected in most proximal tubules but not glomeruli of DN biopsies (Fig. 4I). On the other hand, there was no appreciable difference in PAR-1 and PAR-3 immunostaining between DN and control specimens (Fig. 4F & 4H). Isotype-matched control on DN section (Fig. 4J), non-diabetic control (Fig. 4E) and control omitting the primary antibodies (data not shown) showed negative staining throughout.

**Table 1:** Clinical data and tubular scoring of PARs in diabetic nephropathy patients and non-diabetic control subjects.

Patient type	Age/Genderat biopsy	HbA1C (%)	Serumcreatinine(µmol/L)	Urine proteinexcretion (g)per 24 h	Immunohistochemical staining score*			
					PAR-1	PAR-2	PAR-3	PAR-4
Non-diabetic control group								
1	53/F	7.9	97	<0.03	0	2	1	0
2	47/M	N/A	70	<0.03	0	1	0	1
3	78/F	N/A	71	N/A	1	1	0	1
4	53/M	N/A	114	100 mg/dL	0	1	1	0
5	74/M	N/A	87	<0.03	1	1	1	1
Diabetic nephropathy group								
6	50/M	9.3	227	1.97	0	4	1	4
7	47/M	6.6	844	4.92	1	2	1	3
8	65/M	5.9	750	6.05	2	4	2	2
9	50/M	7.9	215	11.30	1	3	1	2
10	65/F	6.2	284	4.74	1	3	0	2

* quantified on a scoring system 0–5 in which 0 = no cortical staining; 1 = <10% cortical staining; 2 = 10–20% cortical staining; 3 = 20–40% cortical staining; 4 = 40–60% cortical staining; 5 = >60% cortical staining.

### Activation of PAR-4 Induced Pro-inflammatory and Pro-fibrotic Responses in PTEC

**Figure 5:**
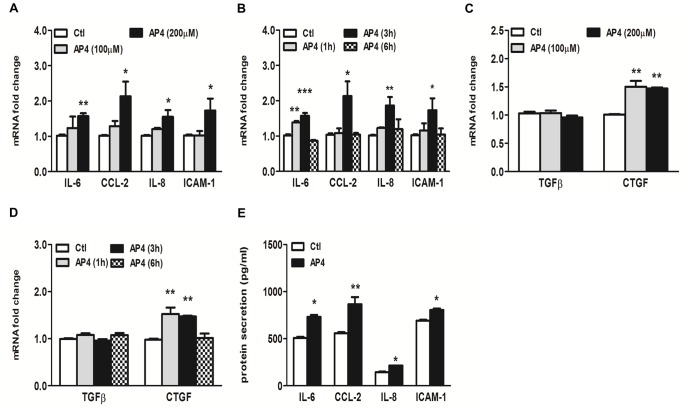
Activation of PAR-4 by selective agonist induced pro-inflammatory and pro-fibrotic responses in PTEC. Cells were incubated with PAR-4 agonist AYPGKF-NH_2_ (AP4) for the indicated duration and doses, and gene and protein expression was determined by real-time PCR and ELISA respectively. PAR-4 agonist increased IL-6, CCL-2, IL-8 and ICAM-1 mRNA expressions in a dose- (A) and time- (B) dependent manner. PAR-4 agonist increased CTGF, but not TGFβ mRNA expression in a dose- (C) and time- (D) dependent manner. PAR-4 agonist induced IL-6, CCL-2, IL-8 and ICAM-1 protein secretion (E). *p<0.05; **p<0.01; ***p<0.001 compared with control.

**Figure 6:**
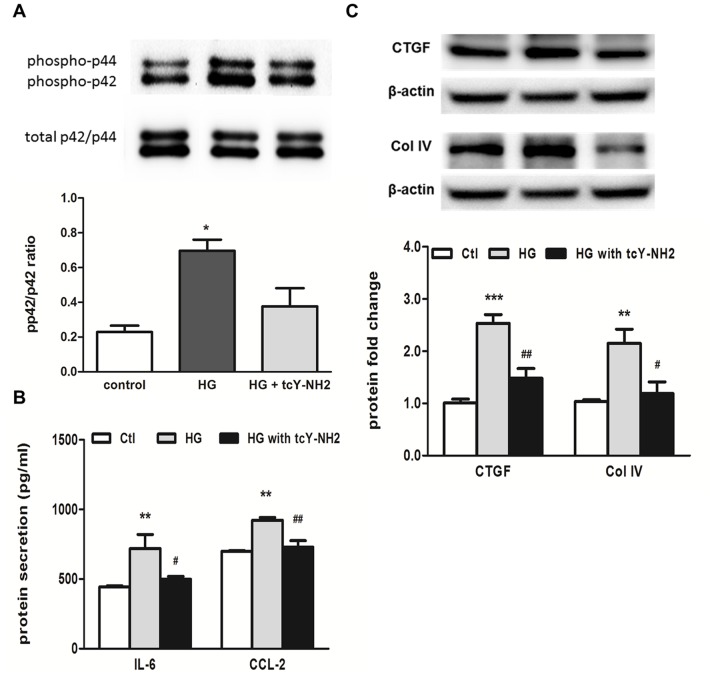
PAR-4 mediated HG-induced IL-6 and CCL-2 expression via p42/44 MAPK signaling in PTEC. Cells were pre-treated with 10 µM selective PAR-4 antagonist, (tcY-NH_2_) for 1 h before treatment with 30 mM HG. Western blot analysis showed an increase in phosphorylated p42/44 MAPK signaling by HG, which was partially inhibited by tcY-NH_2_ (A). HG-induced protein synthesis of IL-6 and CCL-2, detected by ELISA, was attenuated by tcY-NH_2_ (B). HG-induced CTGF and collagen IV up-regulation, detected by Western blot analysis, was attenuated by tcY-NH_2_ (C). *p<0.05; **p<0.01; ***p<0.001 compared with control and ^#^p<0.05; ^##^p<0.01 compared with cells incubated with HG only.

The up-regulation of tubular PAR-4 expression in response to HG led us to investigate whether or not the activation of PAR-4 was implicated in the inflammatory pathway. We first studied the ability of the PAR-4 agonist, AYPGKF-NH_2_, to induce inflammatory response in PTEC. Treatment of PTEC with AYPGKF-NH_2_ resulted in a significant induction of IL-6, CCL-2, IL-8 and ICAM-1 mRNA expression in a dose- and time-dependent manner (Fig. 5A & 5B) by real-time PCR analysis. Maximal up-regulation of all these genes occurred at 3 h after stimulation. Since PAR-1 and PAR-2 activation has been described in the promotion of tissue fibrosis via the production of connective tissue growth factor (CTGF) and thus the accumulation of extracellular matrix (ECM) proteins [31]–[33], we examined the effect of PAR-4 activation on the expression of pro-fibrotic factors. Exposure of PTEC to AYPGKF-NH_2_ did not affect TGFβ mRNA expression, but significantly increased CTGF expression (Fig. 5C & 5D). The increased cytokine secretion was detected in culture medium after 24-h incubation with AYPGKF-NH_2_ (Fig. 5E). To confirm our hypothesis that PAR-4 activation is involved in HG-induced tubular pro-inflammatory and pro-fibrotic pathways, PTEC was pretreated with a PAR-4 antagonist, tcY-NH_2_ prior to HG incubation. Since we and others have shown that induction of IL-6, CCL-2 and CTGF expression by HG are mediated via p42/44 MAPK signaling in human PTEC [9], the expression level of phosphorylated p42/44 protein was determined. Western blot analysis showed that blockade of PAR-4 by PAR-4 antagonist inhibited HG-induced p42/44 phosphorylation (Fig. 6A) and attenuated the subsequent increase in IL-6 and CCL-2 protein synthesis as measured by ELISA (Fig. 6B). In addition, PAR-4 antagonist also inhibited HG-induced CTGF and collagen IV expression (Fig. 6C).

### KLK1 Mediated Pro-inflammatory Responses through PAR-4 Activation

**Figure 7:**
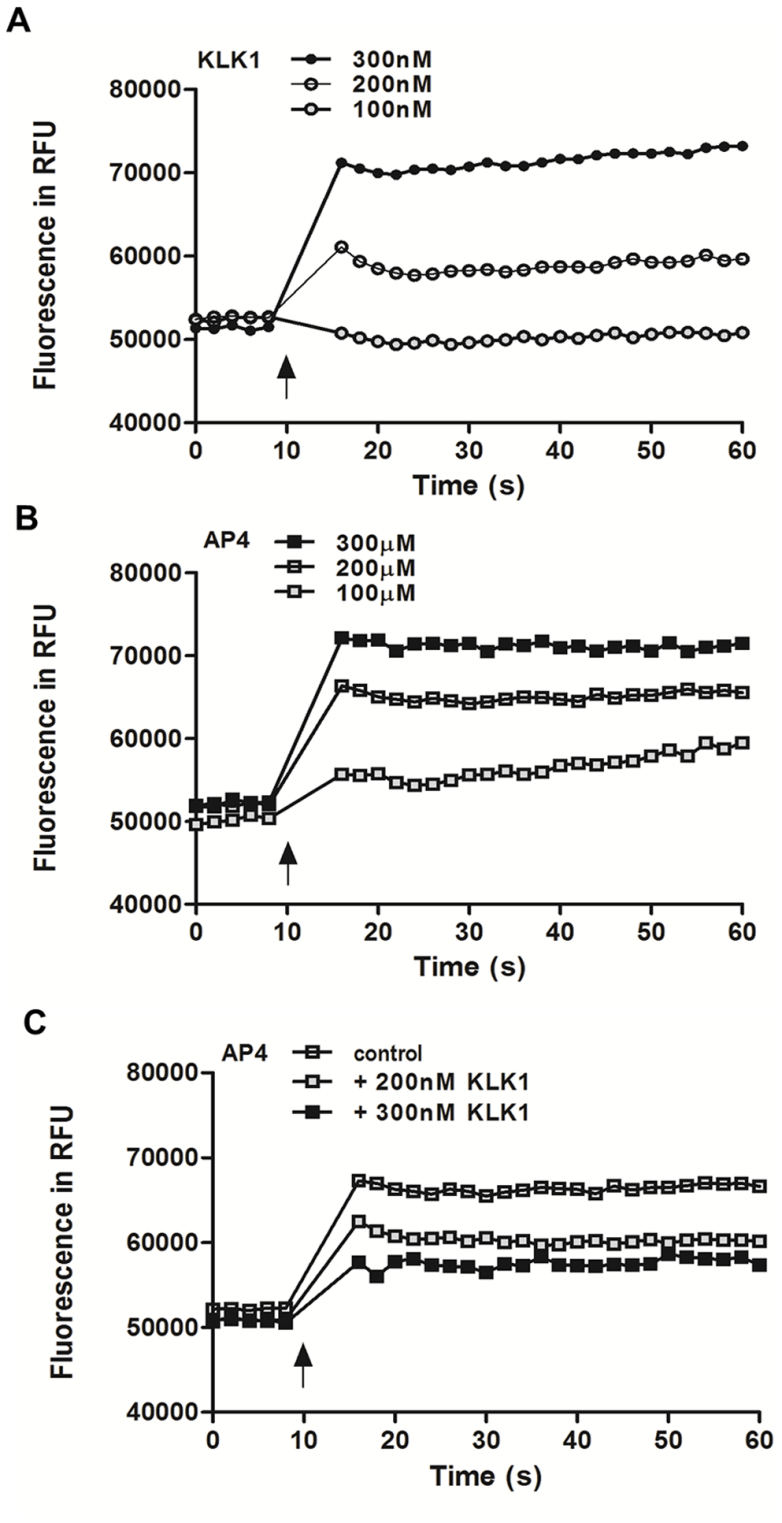
Intracellular calcium mobilization by KLK1 and PAR-4 agonist in PTEC. Cells were incubated with Fluo-4 direct™ reagent solution at 37°C for 1 h and the fluorescence signals induced by the addition of recombinant KLK1 at the indicated concentrations were measured (A). Fluorescence signal induced by the addition of the PAR-4 agonist AYPGKF-NH_2_ (AP4) at the indicated concentrations (B). Fluorescence signal of PAR-4 agonist-induced calcium response in cross desensitization study. Cells were untreated or pretreated with 100 nM or 200 nM KLK1 for 10 min prior to AP4 stimulation (C). (**↑**) indicates the time at which agonist was added. Data are representative of three independent experiments.

**Figure 8:**
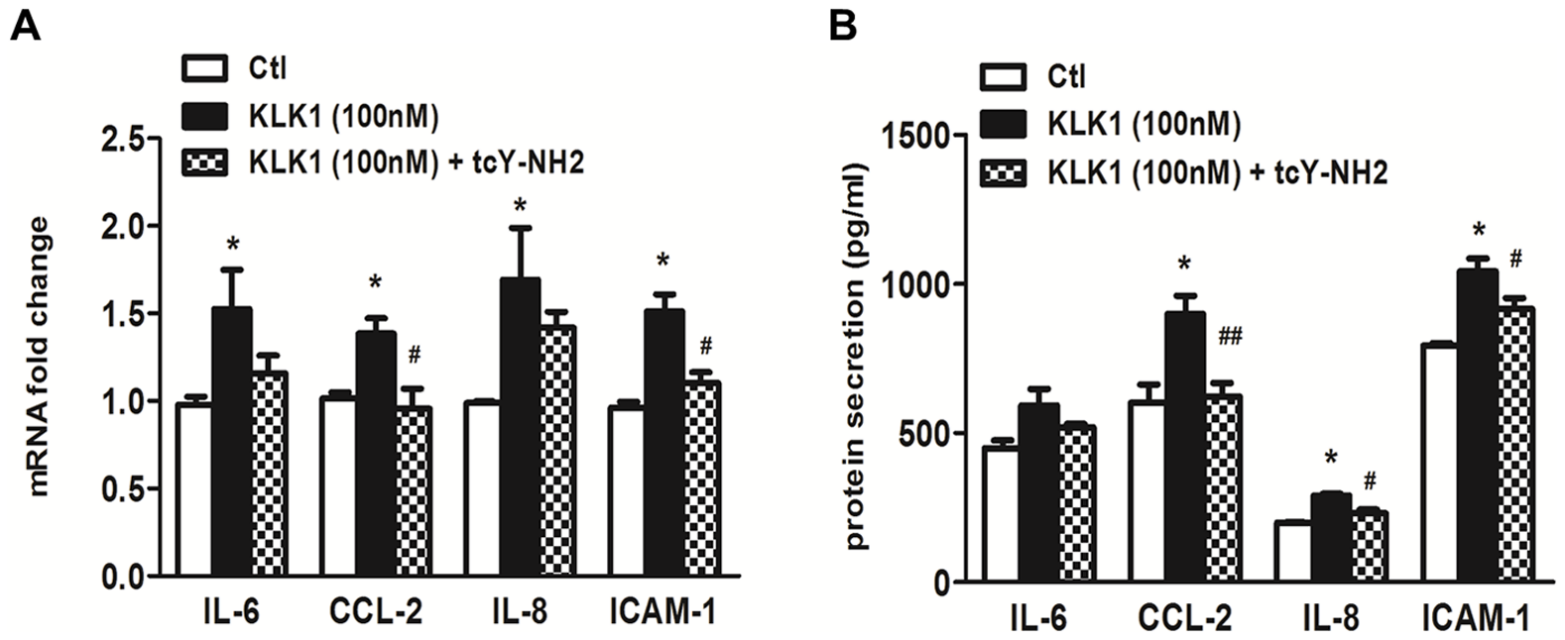
PAR-4 mediated KLK1-induced pro-inflammatory responses in PTEC. Cells were untreated or pre-treated with 10 µM of the selective PAR-4 antagonist, (tcY-NH_2_) for 1 h before treatment with 100 nM KLK1. Gene and protein expression was determined by real-time PCR and ELISA respectively. The increase of KLK1-induced CCL-2 and ICAM-1 mRNA expression was significantly attenuated by PAR-4 antagonist (A) and the increase of KLK-induced CCL-2, IL-8 and ICAM-1 protein expression was significantly reduced by PAR-4 antagonist (B). *p<0.05 compared with control and ^#^p<0.05; ^##^p<0.01 compared with cells incubated with KLK1 only.

Our findings on the up-regulation of PAR-4 by KLK1 suggest the involvement of PAR-4 in KLK1 signaling. Since it has been reported that members of the KLK family can activate PARs by proteolytic cleavage of the N-terminal extracellular domain, and release of a tethered ligand for receptor binding, we next investigated the ability of KLK1 in the activation of PAR-4 using calcium mobilization studies. First, the addition of KLK1 or the selective PAR-4 agonist each elicited a calcium influx response in PTEC in a dose-dependent manner (Fig. 7A & 7B). These data indicate that KLK1 signaling involves an increase in intracellular calcium concentration, and confirm that PAR-4 expressed on PTEC is functional. To determine the ability of KLK1 to activate PAR-4, we performed cross desensitization studies using both KLK1 and the PAR-4 agonist. PTEC pretreated with 100 nM KLK1 for 10 min prior to the challenge of PAR-4 agonist showed a reduced calcium response to PAR-4 agonist at 200 µM. Pretreatment with a higher concentration of KLK1 (200 nM) further attenuated PAR-4 agonist-induced calcium signaling in PTEC (Fig. 7C), confirming that KLK1 activated PAR-4 and desensitized it from the subsequent agonist stimulation. Finally, the role of PAR-4 activation in KLK1-induced pro-inflammatory responses was examined using specific PAR-4 antagonist. As shown in Fig. 8, KLK1-induced CCL-2 and ICAM-1 mRNA and protein expression were significantly reduced by pretreating PTEC with 10 µM PAR-4 antagonist.

## Discussion

Tubulointerstitial inflammation is a critical process in the development of diabetic nephropathy [1], [3], [5]. Increased production of reactive oxygen species, pro-inflammatory cytokines and growth factors by resident and infiltrating cells are associated with the development of tubulointerstitial lesions in both clinical and experimental models of DN [3], [4], [34]. Although we and others have previously shown that bradykinin up-regulated IL-6, CCL-2 and TGF expression in cultured PTEC [9] and the application of aprotinin, the KLK inhibitor, improved renal function of STZ-induced diabetic mice [35], the role of KKS in inflammatory pathways leading to DN is still controversial. Some studies demonstrated a protective effect of KLK1in reducing inflammation, renal fibrosis and glomerular hypertrophy in salt-induced hypertensive rats [36], and improving cardiac function and hypertension in experimental animal models [37], [38].

In the present study, we examined the role of KLK1 in the pro-inflammatory pathway of proximal tubular epithelial cells exposed to the diabetic milieu. Considering that KLK1 expression was induced by HG in cultured PTEC and increased in the proximal tubules of human diabetic kidney tissue [9], we incubated PTEC with recombinant KLK1 and showed that this serine protease caused the activation of p42/44 and p38 MAPK signaling pathways in renal tubular cells and increased the production of inflammatory cytokines, IL-6, CCL-2, IL-8 and ICAM-1, that are relevant to leukocyte recruitment to the interstitial space. Furthermore, knockdown of endogenous KLK1 expression in PTEC inhibited AGE-induced IL-8 and ICAM-1 expression, suggesting that KLK1 mediated the pro-inflammatory responses in diabetic-induced tubular injury.

Differential expression of KLK1 has been identified in several cancers and other diseases [39]. Most of the physiological functions of KLK1 are mediated by kinin receptor B1R and B2R signaling, other studies demonstrated that KLK can participate in direct cell signaling by cleavage and activation of PARs [18], [19], [26]. Here, we showed that KLK1 up-regulated PAR-4 expression, suggesting an interface between the kallikrein-kinin system and coagulation system at the pro-inflammatory pathway of renal tubular cells. The increased PAR-4 expression after KLK1 incubation is consistent with the irreversible nature of PAR activation, in which the activating protease cleaves the extracellular N terminus to expose the tethered ligand, such that a fresh supply of the receptor is required to sustain the action of its agonist [20]. The activation of PAR-4 by KLK1 was ascertained by cross desensitization studies in which cells pretreated with KLK1 showed reduced calcium signaling upon stimulation by the PAR-4 agonist. Furthermore, the pro-inflammatory and pro-fibrotic response triggered by KLK1 was also attenuated when PAR-4 signaling was blocked. Taken together, these results suggest that KLK1 mediates tubular inflammation through PAR-4 activation.

The participation of PAR in DN was further demonstrated by the up-regulation of PAR-2 and PAR-4 protein in human diabetic kidney tissue. Increased PAR-2 and PAR-4 expression were detected mostly in tubular cells and little expression was found in glomerular areas. Increased renal PAR-2 expression was previously reported in the infiltrating cells and proximal tubuli of patients with IgA nephropathy [22] as well as in the glomeruli of diabetic db/db mice [33]. PAR-2 is a potent pro-inflammatory mediator in keratinocytes [40] and kidney cells [33], [41], [42]. PAR-2 activation also triggers angiogenesis that contributes to tumor growth and wound healing. However, few studies have examined PAR-4 since the expression of this receptor is barely detectable in many cell types. Here, we describe for the first time a markedly increase in expression of PAR-4 after HG stimulation in PTEC and in human diabetic kidney tissue, compared to PAR-1 and PAR-2 expression, suggesting a role of PAR-4 in the pathogenesis of DN.

Both thrombin and trypsin stimulate pro-inflammatory responses via the activation of PAR in primary culture of human PTEC [41], [43], but not all the effects of thrombin could be reproduced by the PAR-1 agonist, implying that other family members may be involved in provoking these inflammatory responses in PTEC. Several groups have reported the pro-inflammatory effect of PAR-4 activation in endothelial cells [44], [45], neutrophils [30] and sensory neurons [46], and suggested that PAR-4 may play an important role in the early event of inflammation including leukocyte rolling and adhesion process [30], [45], [47]. Our data not only revealed the cytokine-releasing function of PAR-4 in the proximal tubular cells, but also demonstrated a PAR-4 mediated pro-inflammatory pathway in response to HG stimulation. PAR-4 antagonist blocked HG-induced p42/44 MAPK phosphorylation in PTEC and attenuated the downstream induction of pro-inflammatory cytokines (IL-6 and CCL-2), pro-fibrotic factor (CTGF) and collagen IV synthesis, indicating the involvement of PAR-4 in this process via the activation of MAPK signaling.

Although both PAR-1 and PAR-4 are thrombin receptors, the up-regulation of PAR-1 protein in the diabetic kidney is not significant by immunohistochemical staining. This may be due to the difference in receptor potencies and kinetics of desensitization. PAR-1 responds to low enzyme concentration and mediates rapid and transient activation, whereas PAR-4 only responds to high enzyme concentration and causes a delayed and sustained activation [20]. As a result, up-regulation of PAR-4 expression may become more significant in prolonged stimulation as diabetic nephropathy progress. In addition, PAR-2 is often co-expressed with PAR-4 as they are both up-regulated by tumor necrosis factor-α. A recent study also revealed the heterodimerization between PAR-2 and PAR-4 proteins, suggesting the regulatory role of PAR-2 in the membrane trafficking of PAR-4 [48].

**Figure 9:**
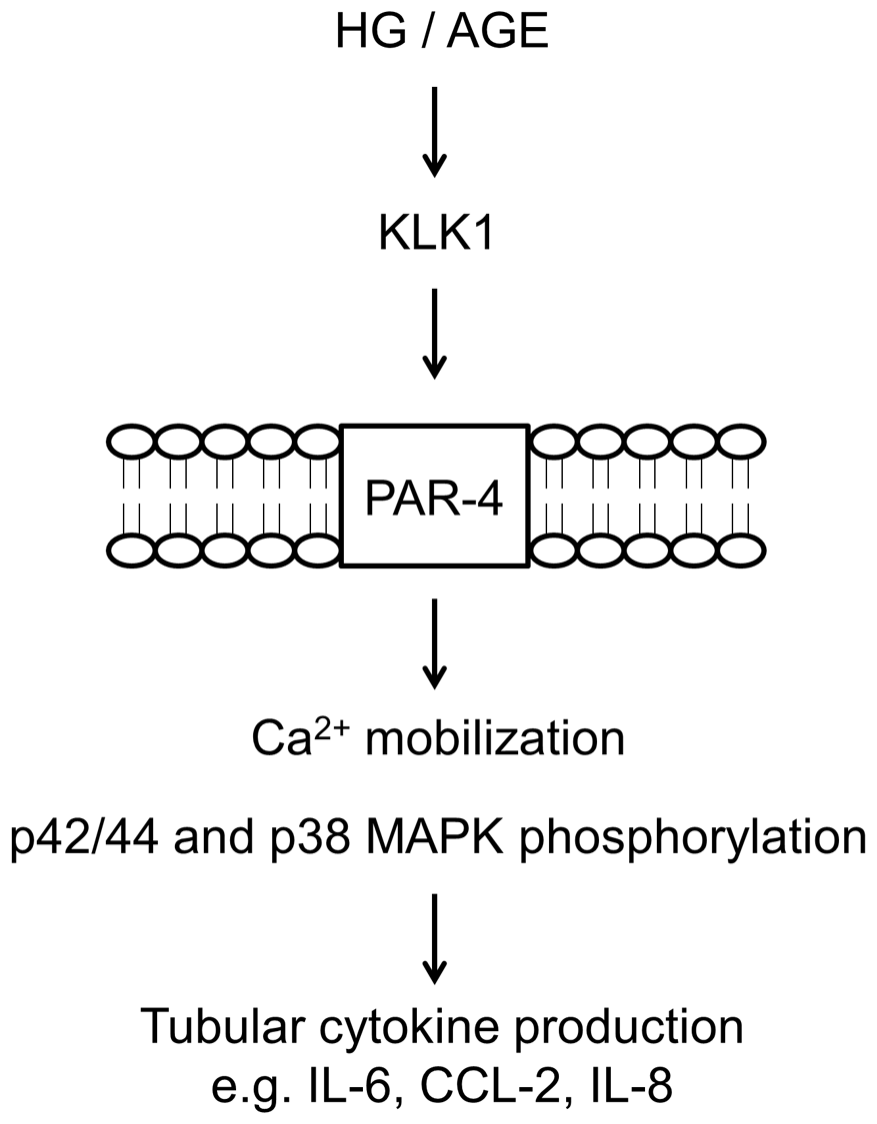
Schematic diagram illustrating the proposed activation of PAR-4 signaling by KLK1 in tubular inflammation. Under the diabetic milieu, HG or AGE induces the expression of KLK1, which leads to PAR-4 activation, intracellular Ca^2+^ mobilization and phosphorylation of MAPK signaling, and results in subsequent cytokine production.

To summarize, our results demonstrate for the first time the pro-inflammatory effect of KLK1 on PTEC. As illustrated in Fig. 9, it is likely that HG and AGE upregulate the expression of KLK1, which is a serine protease capable of activating PAR-4, leading to intracellular calcium mobilization, phosphorylation of MAPK, and the subsequent cytokine synthesis. In fact, we propose that KLK1 promotes tubular inflammation through the activation of multiple receptors. In most studies, KLK1 targets B2R via the formation of bradykinin (BK) [49] and we previously demonstrated this phenomenon in HG stimulated PTEC [9] although KLK1 was recently shown to directly activate B2R in the absence of kininogen both *in vitro* [50] and *in vivo* [51] in other cell types. However, application of B2R blocker, icatibant only partially reduced HG-induced inflammatory responses and protected the diabetic db/db mice from renal damage [9], [52]. These data support our hypothesis that multiple pathways are involved in the inflammatory process under the diabetic condition. Interestingly, recent studies from a rat paw inflammation model showed that edema induced by PAR-4 agonist was blocked by B2R antagonist [53], and PAR-4-induced sensitization of rat joint primary afferents involved B2R activation [54], suggesting a crosstalk between PAR-4 and B2R, but the mechanism remains unknown and further investigation is needed to dissect the possible association between these receptors in other cell types. In conclusion, this study suggests a novel pathway in which KLK1 plays a role in tubular inflammation via the activation of PAR-4 and provides a potential therapeutic target for DN in the future.
